# Heme-heme oxygenase-2 reduces the atherosclerosis by preventing inflammation

**DOI:** 10.1016/j.crphar.2022.100141

**Published:** 2022-12-12

**Authors:** Zhenzhen Wang, Xiaoqiang Zhan, Shuai Yang, Yang Chen, Yingchao Bi, Xuemei Xian, Quangang Chen, Xufeng Han, Zhangping Yang, Renjin Chen

**Affiliations:** a Cancer Institute, Xuzhou Medical University, Xuzhou, Jiangsu, China; b College of Life Sciences, Xuzhou Medical University, Xuzhou, Jiangsu, China; c Jiangsu Key Laboratory of Animal Genetic Breeding and Molecular Design, Yangzhou University, Yangzhou, Jiangsu, China

**Keywords:** Heme oxygenase-2, Atherosclerosis, Macrophages, Inflammation

## Abstract

**Objective:**

Heme oxygenase (HO) has been shown to have important antioxidant and anti-inflammatory properties, resulting in a vascular antitherogenic effect. This study was undertaken to evaluate the role of HO-2 in atherosclerosis.

**Method and results:**

The expression levels of HO-2 were evaluated in M1 and M2 bone marrow macrophage induced by LPS and IL4. The expression of HO-2 was significantly higher in M2 macrophage than in M1 macrophage. Western diet (WD) caused a significant increase in HO-2 expression in ApoE^−/−^ mice. The adeno-associated viral (AAV) vectors expressing HO-2 was constructed, and the mice were received saline (ApoE^−/−^), AAV (ApoE^−/−^), AAV–HO–2 (ApoE^−/−^) on WD at 12 weeks and their plasma lipids, inﬂammatory cytokines, atherosclerosis were evaluated for 16 weeks. The results showed AAV–HO–2 was robust, with a significant decrease in the en face aortas, lipids levels, inﬂammatory cytokines and M1 macrophage content in AAV–HO–2 ApoE^−/−^ compared to control AAV-ApoE^−/−^.

**Conclusion:**

HO-2 expression in macrophages plays an important role of the antiatherogenic effect, decreasing the inflammatory component of atherosclerotic lesions. These results suggest that HO-2 may be a novel therapeutic target for cardiovascular diseases.

## Highlights

• Heme-heme oxygenase-2 can promote the M2 macrophage phenotype.
• AVV–HO–2 significantly inhibit the formation and development of arteriosclerosis.
• HO-2 expression decreased atherosclerotic lesions, proinflammatory cytokines and lipid level.

## Graphical abstract

**Figure:**
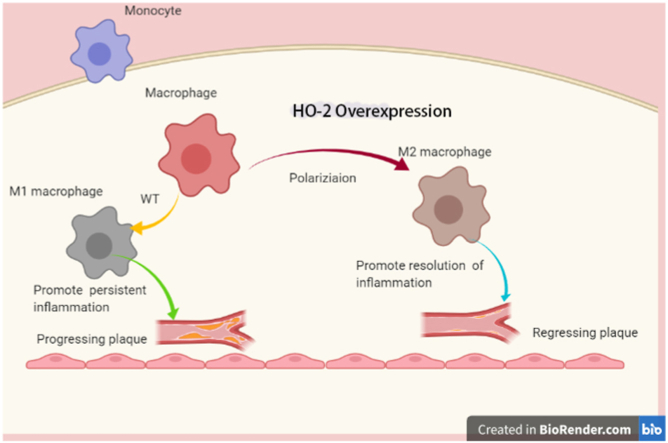

## 1 Introduction

Atherosclerosis is a chronic inﬂammatory disease that is a leading cause of morbidity and mortality worldwide (Rosamond et al., 2008). Macrophages play an important role in atherosclerosis by attempting to clear the inflammatory lipids and then being transformed into cholesterol-loaded macrophages or foam cells (Koelwyn et al., 2018). These macrophage foam cells persist accumulation in the aortic wall, leading to the progression of atherosclerotic lesion (Weber and Noels, 2011). The atherosclerotic microenvironment is very important.

Heme oxygenase (HO) is a rate-limiting and microsomal enzyme that cleaves heme into equimolar amounts of iron, carbon, monoxide, and biliverdin (Willoughby et al., 2000; Yoshida and Kikuchi, 1978). Its subsequent metabolites of heme catabolism seem to play vital roles in regulating important biological responses including inflammation, oxidative stress, cell survival, and cell proliferation. HO has three isoforms: HO-1 is an inducible of HO, HO-2 is constitutively expressed, and HO-3 is nearly devoid of catalytic activity (Abraham and Kappas, 2008). HO-1 is transcriptionally upregulated by oxidized LDL as a sensitive anti-inflammatory protein in vascular endothelial cells, vascular smooth muscle cells, and macrophages (Wang et al., 1998). HO-1 has shown to be a novel therapeutic target for cardiovascular diseases (Morita, 2005). However, HO-2 is also expressed in macrophages and the question about whether HO-2 is associated with atherosclerosis in apoE null (ApoE^−/−^) mice is poorly known. We hypothesized that macrophage HO-2 acts as a sensitive anti-inflammatory protein to protect against the development of atherosclerosis.

## 2 Material and methods

**Fig. 1:**
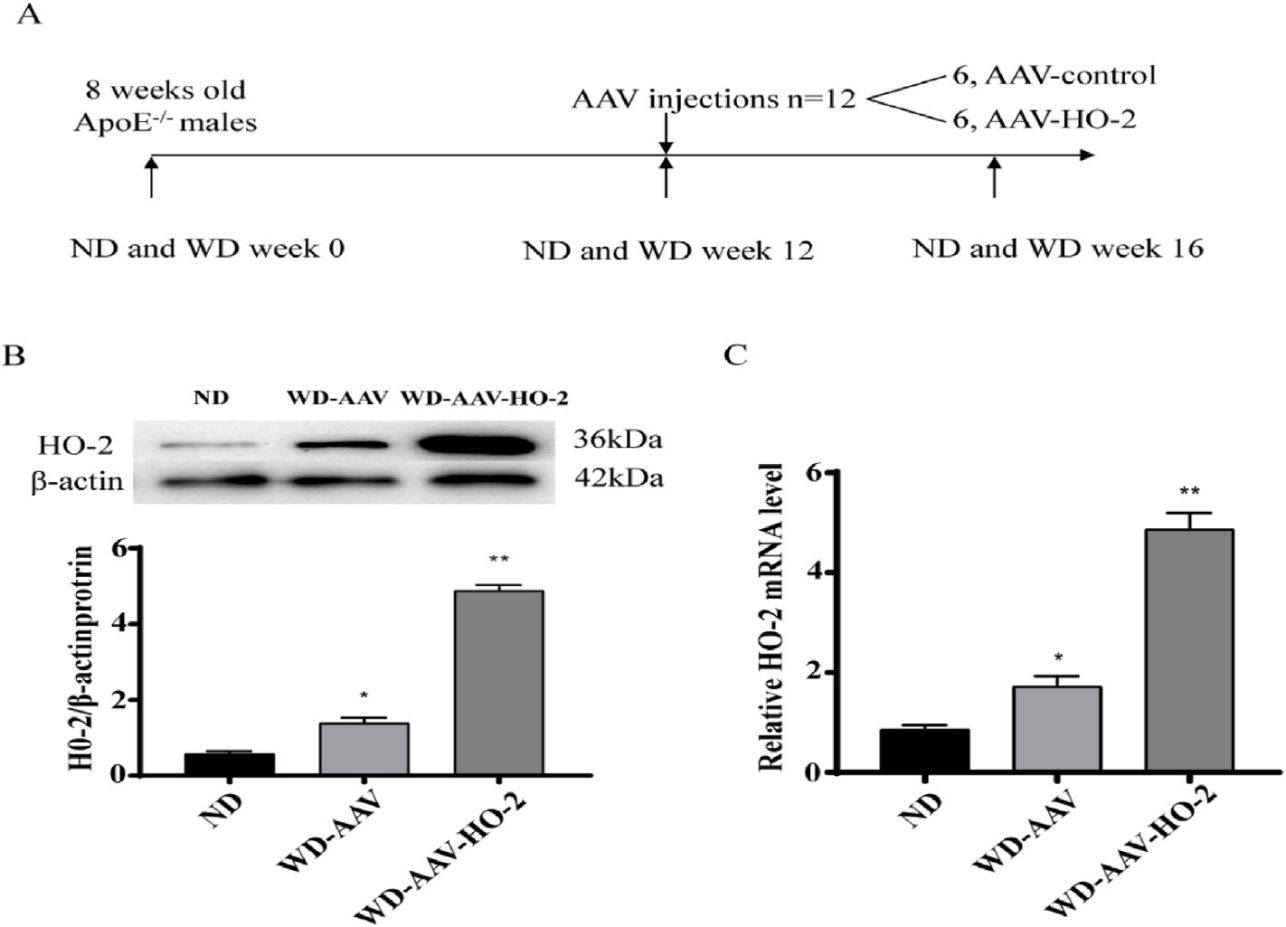
The level of expression of HO-2 in whole aortas of ApoE^−/−^after AVV–HO-2 infection. ApoE^−/−^ were fed normal diet (ND), Western diet (WD) for 12 weeks, then transduced with AVV–HO–2 and AVV, so the groups are ND, WD-AVV and WD-AVV–HO–2. A, Scheme of experiment. B, The protein expression levels were measured by Western blot (n ​= ​3). C, The mRNA expression levels of HO-2 were measured by qPCR in whole aortas (n ​= ​3); Data (B, C are shown as the mean ​± ​SD of two independent experiments performed in triplicate. Values of qPCR and protein expression were normalized to β-actin. ∗∗ AVV–HO–2 vs WD-AAV, *P* ​< ​0.01, ∗ WD-AAV vs ND, *P* ​< ​0.05. *P* ​< ​0.01, *P* ​< ​0.05 by one-way ANOVA with Tukey's multiple comparisons test; deviation bars indicate standard deviation of the mean.

**Animal model.***ApoE*^*−/−*^ mice were obtained from the Model Animal Center of Nanjing University. The mice were divided into three cohords: *ApoE*^*−/−*^(6) with normal diet (ND) (20% kcal protein, 16% kcal fat, 18% kcal carbohydrate), *ApoE*^*−/−*^(6) with Western diet (WD) (20% kcal protein, 40% kcal fat, 40% kcal carbohydrate), and *ApoE*^*−/−*^ (AAV)（6）with Western diet (WD) (20% kcal protein, 40% kcal fat, 40% kcal carbohydrate). Males were used in the study. Mice were fed normal diet (ND) and Western diet (WD) from 4 weeks of age for 16 weeks and then euthanized, and peripheral blood, heart and aorta were harvested (Fig. 1 A). ApoE^*−/−*^ mice with WD were treated with AAV–HO–2 at 12 weeks. Bone marrow-derived macrophages were harvested from C57BL/6 mice. All mice were retained in a pathogen-free facility of Xuzhou Medical University. Animal experimental procedures were performed in accordance with the ethical guidelines of Xuzhou Medical University (Xuzhou, China).

### 2.1 AAV–HO–2 production and delivery

The serotype 9-based AAV vector, which was driven by CMV promotor, was constructed and murine cDNA HO-2 was cloned into AAV 9 vector. The AAV–HO–2 vector viruses were packaged using HEK 293T cells using the triple transfection method. Viruses were purified by a single cesium chloride gradient. The viral stock was tittered by dot-blot hybridization with plasmid standards. AAV-NC served as the control for AAV–HO–2. ApoE^*−/−*^ mice were fed WD for 12 weeks, which were injected with AAV–HO–2 or AAV-NC on WD for 4 weeks of study. 3.5 ∗ 10^11^ vg/mL/mouse was injected by tail vein.

### 2.2 Oil Red O staining

The aortas analysis: the aortas were isolated after PBS perfusion, split and pinned onto a silicone plate in a “Y” shape, then fixed with 4% paraformaldehyde for 60 ​min, washed three 3 times with PBS for 5 ​min each and incubated in 60% isopropanol for 2 ​min followed by incubation in 0.5% Oil Red O solution for 1 ​h. De-staining was performed with 75% ethanol, then washed three 3 times with tap water each. Imaging of aortas was performed using a Leica stereomicroscope. Quantification of lesions was performed by manually tracing the aorta and lesion areas with Image-Pro Plus 6.0 software. For analysis of lesions in the aortic sinus, the cryosections were washed 3 times with PBS for 5 ​min each and incubated in 60% isopropanol for 2 ​min followed by incubation in 0.5% Oil Red O solution for 10 ​min. The consistency of lesion size in the aortic root was measured by quantifying sections at intervals of 80 μm using Image-Pro Plus 6.0 software.

**Immunofluorescent staining**. Mouse aortic root was embedded in OCT media and sectioned at a thickness of 5 ​μm. The sections were air-dried at room temperature for 30 ​min, washed 3 times in 1 ​× ​PBS (pH7.4) for 5 ​min each, fixed with 4% paraformaldehyde for 10 ​min and then permeabilized with 0.25% Triton X-100 for 5 ​min. Next, sections were blocked with 1% BSA (prepared in 1 ​× ​TBST, pH 7.4) for 1 ​h, and then incubated with primary antibodies (CD206, 18704, from Proteintech Group, 1:100 immunofluorescent dilution; HO-2, ab-90492, from abcam, 1:1000 immunofluorescent dilution) overnight at 4 ​°C, followed by incubation with fluorophore-conjugated secondary antibodies for 1 ​h at room temperature. Nuclei were labeled with DAPI. The stained cells were visualized and photographed with an Olympus fluorescence inverted microscope at a magnification of ​× ​200. and positive results were quantified using ImageJ software.

**Cell culture.** The bone marrow-derived macrophages cells （BMDMs）were isolated and cultured in DMEM containing 10% FBS and 1% PennStrep at 37 ​°C with 5% CO_2_. The cells were stimulated with lipopolysaccharide (100 ​ng/mL) or interleukin 4 (20 ​ng/mL) for 24 ​h for Western blots and qPCR.

**RNA isolation and quantitative real-time PCR**. Total RNA was extracted from cells with Trizol reagent (Invitrogen) and reverse transcribed using SuperScriptII (Invitrogen). RT-PCR analysis was conducted on Roche a 480 real-time detection system using SYBR Green PCR Master Mix reagent. Moreover, the expression levels of the target genes were presented as the mean ​± ​SD normalized to β-actin expression adopting the ΔΔCt method. Primers are listed in Table 1.

**Table 1:** Primers for qPCR.

Gene	Primer sequence (5′to3′)
eIF6	Forward: AGAGCGTCGTTCGAGAACAAC
	Reverse: CGGGAATGGCATCGGAGAG
IL-1β	Forward: GAAGAAGAGCCCATCCTCTG
	Reverse: TCATCTCGGAGCCTGTAGTG
IL-6	Forward: AGTCCGGAGAGGAGACTTCA
	Reverse: TTCCACGATTTCCCAGAG
iNOS	Forward: CCCTTCCGAAGTTTCTGGCAGC
	Reverse: GGCTGTCAGAGCCTCGTGGCTTTGG
MCP-1	Forward: GAAGGAATGGGTCCAGACAT
	Reverse: ACGGGTCAACTTCACATTCA
TNF-α	Forward: CCTGTAGCCCACGTCGTAG
	Reverse: GGGAGTAGACAAGGTACAACCC
Arg1	Forward: TGATGTTGACGGACTGGACC
	Reverse: ATCTAATCCTGAGAGTAGCCCTGT
CD163	Forward: GGGTCATTCAGAGGCACACTG
	Reverse: CTGGCTGTCCTGTCAAGGCT
TGF-β1	Forward: CCGCAACAACGCCATCTATG
	Reverse: CTCTGCACG GGACAGCAAT
GAPDH	Forward: CAACTTTGGCATTGTGGAAGG
	Reverse: ACACATTGGGGGTAGGAACAC

**Western blotting**. The proteins were extracted from the bone marrow-derived macrophages cells and en face aortas, and their concentrations were determined by the BCA method. The membranes were incubated with each primary antibody (Ab) as follows: HO-2 antibodies (ab-90492; 1:1000 western blotting dilution), incubated at 4 ​°C overnight, followed by incubation with the appropriate horseradish peroxidase-conjugated secondary antibodies β-actin (1:1000) at room temperature for 2 ​h. The immunoreactive proteins were detected by enhanced chemiluminescence with autoradiography.

Finally, the quantification of bands was performed by densitometric analysis using ImageJ software (Free Software Foundation Inc., Boston, MA, USA).

**Analysis of lipid levels of cellular and serum.** Plasma lipid profles of total cholesterol (TC), triglyceride (TRG), HDL cholesterol (HDLc), and LDL cholesterol (LDLc) were measured by enzymatic colorimetric kits according to the manufacturer's protocol (Nanjing Fuji Bioengineering Institute, Nanjing, China).

**Flow cytometry analysis.** Flow cytometry of aortas: whole aortas were isolated from mice on Western diet for 16 weeks. Aortas were dissected, homogenized, and placed in an enzyme solution containing hyaluronidase type I–S (60 U/mL), DNase I (1 U/mL) and collagenase type I in 10 ​mM Hepes, shaking at 37 ​°C for 60 ​min. Cells were put through a 200-mesh filter and rinsed with PBS, and then stained with antibodies against CD45, anti-CD11b, anti-F4/80, anti-CD86, anti-CD206 for 20 ​min for the different cell population. Data analysis was performed using FlowJo software (TreeStar Inc., Ashland, OR, USA) to exclude cell debris, clustered cells and dead cells by gating.

**Statistical analysis.** Statistical approach of bioinformatic analyses is described in the appropriate sections above. Using GraphPad Prism Software, p ​< ​0.05 was considered statistically significant. Values are presented as means ​± ​standard deviation (SD), Differences between two groups were assessed by unpaired two-tailed Student's t-test, and the comparison between more than two groups was performed by a one-way analysis of variance (ANOVA).

## 3 Results

### 3.1 The expression changes of HO-2 in M1 and M2 macrophages and effect of HO-2 on lipids, atherosclerotic lesions

The bone marrow-derived macrophages were obtained to evaluate the express changes of HO-2 induced by LPS (100 ​ng/mL) and IL4 (20 ​ng/mL). The mRNA and protein express levels of HO-2 were measured by Real-time PCR and Western blotting. The mRNA and protein express levels of HO-2 induced by IL4 were significantly higher than those induced by LPS and control (Fig. S1 A. B).

The mRNA and protein express levels of HO-2 were measured by real-time PCR and Western blotting in the whole aortas of ApoE^−/−^ and control mice on Normal diet and Western diet for 16 weeks. The results showed that WD group up-regulated HO-2 expression in aorta compared with the ND group (Fig. S1 C. D).

We used the AAV–HO–2 system to overexpress HO-2 stable in the whole aorta. Firstly, we detected infection efficiency of AAV. ApoE^−/−^ mice were injected with AAV at 4 weeks of age, and evaluated at 8 weeks. The mRNA and protein levels of HO-2 were significantly increased in the ApoE^−/−^ -AVV–HO–2 compared with the ApoE^−/−^-AAV at that time (Fig. 1 B. C).

**Fig. 2:**
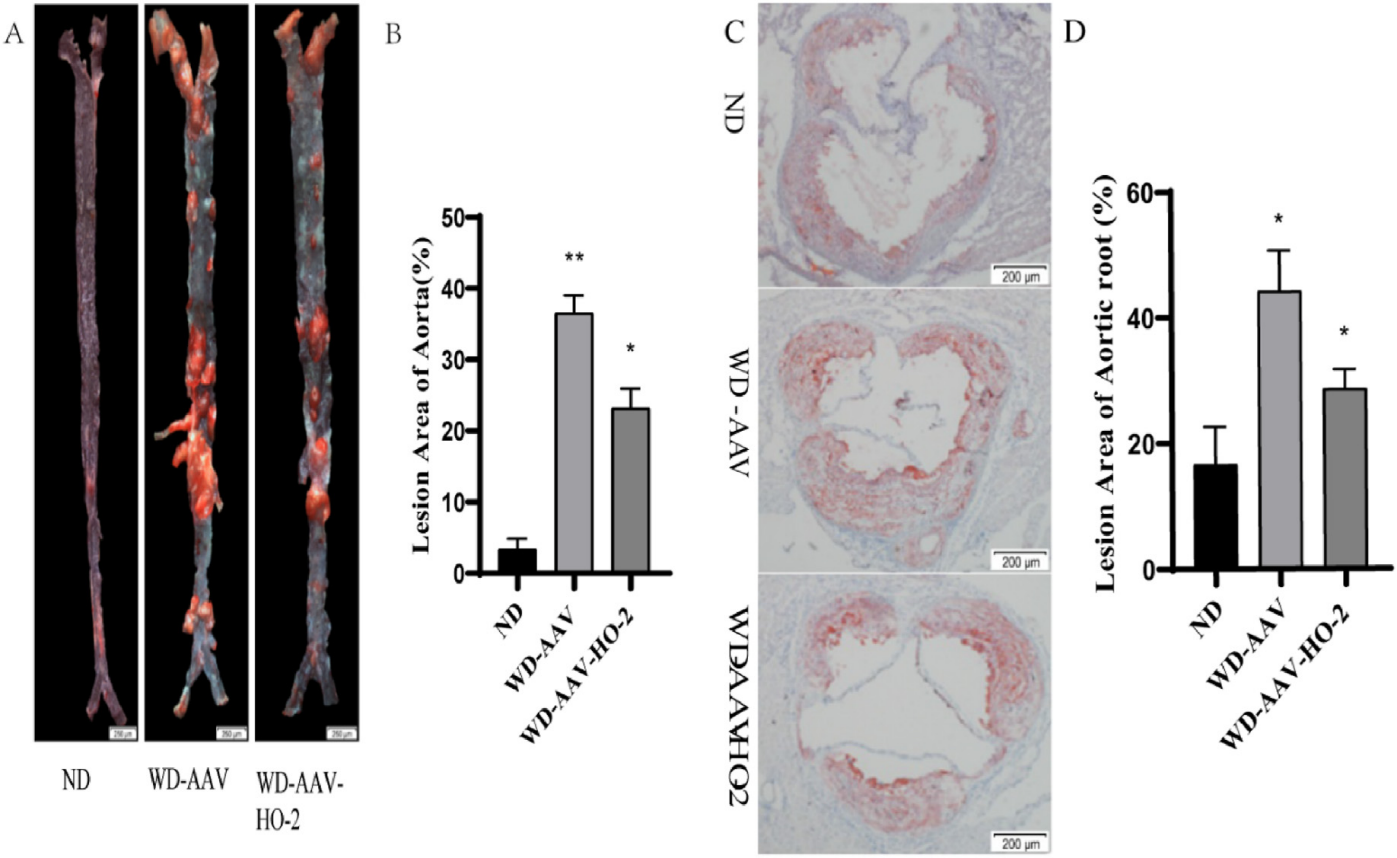
Whole aortas and aortic root sections were dissected and stained with Oil Red O. A. C, Representative en face aortas and aortic root of Oil Red O. Scale bar ​= ​2 ​mm. B. D, Quantification of Lesion area of aorta in normal diet (ND), Western diet (WD) and Western diet (WD)-AAV–HO–2. Scale bar ​= ​200 ​μm. Data (B–D) are shown as the mean ​± ​SD of two independent experiments performed in triplicate. ∗ WD-AAV vs WD-AVV–HO–2, ∗ WD-AAV–HO–2 vs ND, *P* ​< ​0.05. *P* ​< ​0.01, *P* ​< ​0.05 by one-way ANOVA with Tukey's multiple comparisons test; deviation bars indicate standard deviation of the mean. (For interpretation of the references to color in this figure legend, the reader is referred to the Web version of this article.)

**Fig. 3:**
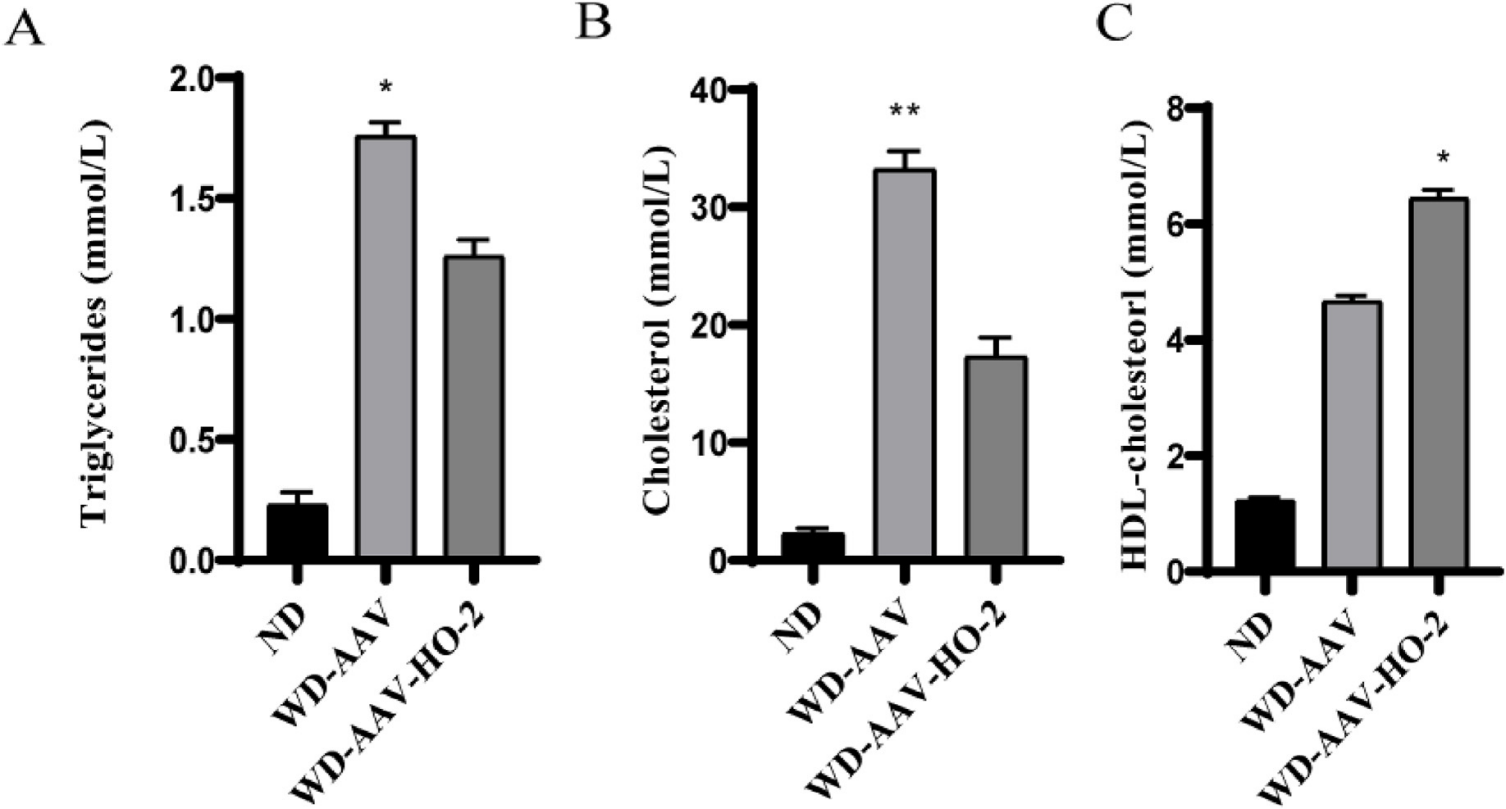
Serum triglyceride, cholesterol, and DHL-cholesterol levels in ApoE^−/−^ and ApoE^−/−^/AAV–HO–2 mice fed normal diet (ND; 6) and western diet (WD; 6) for 16 weeks. A. B. C, The plasma levels of triglyceride, cholesterol and DHL-cholesterol were measured by gas chromatography (GC/MS). Data (A–C) are shown as the mean ​± ​SD of two independent experiments performed in triplicate. ∗ WD-AVV–HO–2 vs WD-AAV, *P* ​< ​0.05, ∗∗ WD-AVV–HO–2 vs WD-AAV, *P* ​< ​0.01. *P* ​< ​0.01, *P* ​< ​0.05 by one-way ANOVA with Tukey's multiple comparisons test; deviation bars indicate standard deviation of the mean.

To analyze the effect of overexpress HO-2 on atherosclerotic lesions, lipids and total cholesterol levels were measured. The whole aortas and aortic root of mice were stained with Oil Red O, and the lesion areas in WD-AAV mice were relatively higher compared with the WD-AVV–HO–2 mice. And the lesion areas in WD-AVV–HO–2 mice were higher compared with ND mice. (Fig. 2 A. B. C. D). The total cholesterol and triglyceride levels were significantly reduced in the AAV–HO–2 treated mice (Fig. 3 A. B.) compared with the control mice. But the HDL-cholesterol levels in the AAV–HO–2-treated mice were increased compared with the control mice (Fig. 3 C).

### 3.2 HO-2 promotes an anti-inﬂammatory macrophage phenotype

**Fig. 4:**
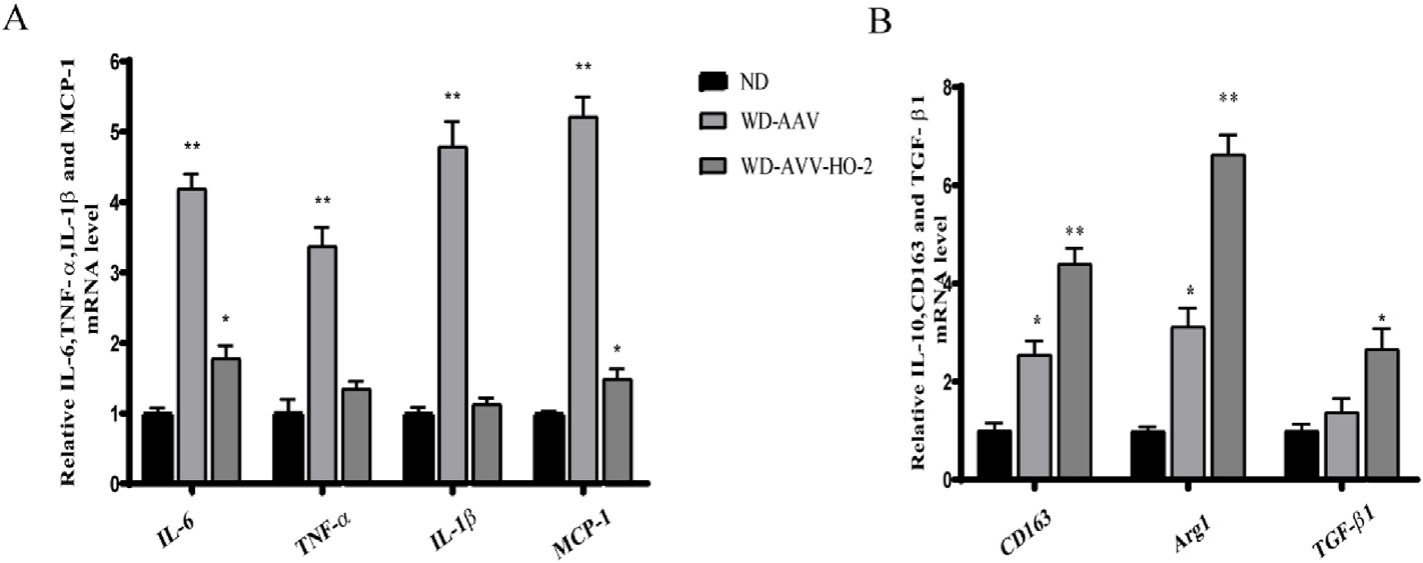
AVV–HO–2 increased the expression of anti-inﬂammatory factors and reduced pro-inﬂammatory factors. A. B, The inflammatory cytokines expressions levels were measured by qPCR in the aortic sinus of ApoE^−/−^ (ND, 6; WD; n ​= ​6) and ApoE^−/−^/AVV–HO–2 (WD; n ​= ​6) fed ND and WD for 16 weeks for 16 weeks. Data (A–B) are shown as the mean ​± ​SD of two independent experiments performed in triplicate. (A, ∗∗WD-AVV–HO–2 vs WD-AAV, *P* ​< ​0.01; ∗WD-AVV–HO–2 vs ND, *P* ​< ​0.05; B, CD163, Arg1, ∗∗ WD-AVV–HO–2 vs WD-AAV, *P* ​< ​0.01; ∗ WD-AVV–HO–2 vs ND, *P* ​< ​0.05; TGF-β1, ∗ WD-AVV–HO–2 vs WD-AAV, *P* ​< ​0.05). *P* ​< ​0.01, *P* ​< ​0.05 by one-way ANOVA with Tukey's multiple comparisons test; deviation bars indicate standard deviation of the mean.

To further verify that AAV–HO–2 affects the phenotype of macrophages in vivo, the en face aortas of the AAV–HO–2-treated mice and the control mice were collected at 16 weeks. Proinﬂammatory cytokines (TNFα, iNOS, IL1β, IL6 and MCP-1) and anti-inflammatory cytokines (TGF-β1, CD163 and Arg1) were detected by real-time PCR in en face aortas. The results showed that HO-2 increased the expression of anti-inﬂammatory factors and reduced pro-inﬂammatory factors in the whole aortas of the ApoE^−/−^-AAV–HO–2-treated mice and compared with the ApoE^−/−^-AAV (Fig. 4 A. B).

**Fig. 5:**
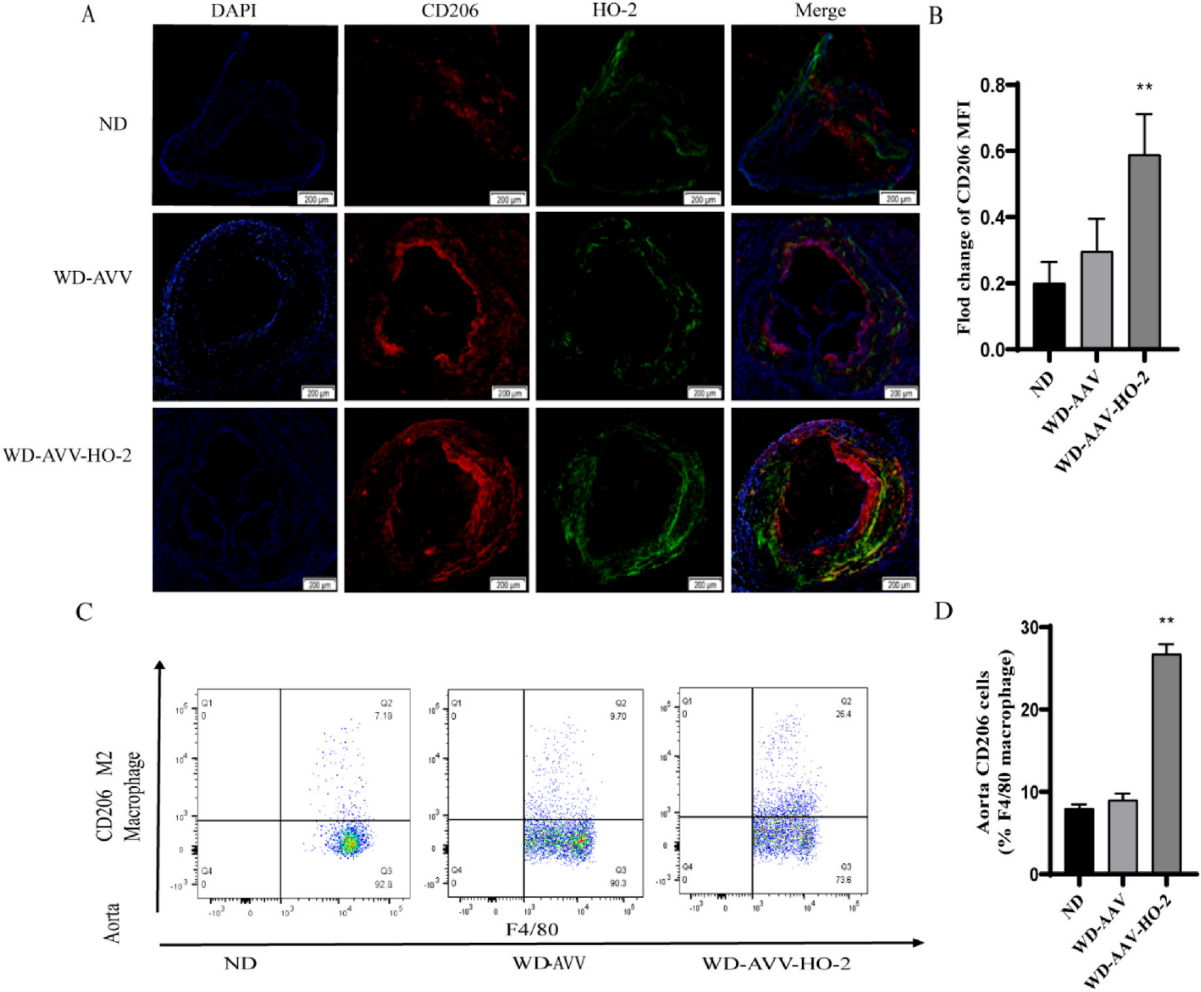
AVV–HO–2 promotes an anti-inﬂammatory macrophage phenotype. A, Aortic root sections from in ApoE^−/−^ and AVV–HO–2/ApoE^−/−^ mice fed normal diet (ND; 6) and western diet (WD; 6) for 16 weeks were stained for DAPI (blue), CD206 (red) and HO-2 (green), Scale bar ​= ​200 ​μM. B, The M2 macrophage (CD206) content in WD-AVV–HO–2 mice were significantly higher than ND and WD-AAV mice. Data are shown as the mean ​± ​SD of two independent experiments performed in triplicate. ∗∗ WD-AVV–HO–2 vs WD-AAV, *P* ​< ​0.01. C, Gating strategy for flow cytometric analysis of anti-inflammatory macrophage M2 (CD206) populations of in ApoE^−/−^ (ND, 6; WD, 6) and ApoE^−/−^/AVV–HO–2 (WD, 6) fed ND and WD for 16 weeks. (The major leukocyte population was selected in forward versus side scatter plots and single cell determination was performed, live CD45^+^ cells were then selected, the macrophage was CD11b+; F4/80+, and the anti-inflammatory population of macrophage population was further gated to determine CD206+ cells). D, M2 (CD206) populations in WD-AVV–HO–2 mice were significantly higher than ND and WD-AAV mice. Data are shown as the mean ​± ​SD of two independent experiments performed in triplicate. ∗∗ WD-AVV–HO–2 vs WD-AAV, *P* ​< ​0.01. *P* ​< ​0.01, *P* ​< ​0.05 by one-way ANOVA with Tukey's multiple comparisons test; deviation bars indicate standard deviation of the mean. (For interpretation of the references to color in this figure legend, the reader is referred to the Web version of this article.)

To further analyze the effect of AAV–HO–2 on phenotype macrophages, the proportion of M2 macrophages were analyzed in the whole aortas of the AAV–HO–2-treated mice and the control mice fed WD via immunoﬂuorescent staining. The results showed that the AAV–HO–2-treated mice had significantly increased M2 macrophage content compared with the control mice (Fig. 5 A. B). We further analyzed macrophage content of the aortic root in the AAV–HO–2-treated mice and the control mice via ﬂow cytometry. The M2 macrophage (CD206) content of the aortic root was increased via immunoﬂuorescent staining in the ApoE^−/−^-AAV–HO–2 transduced mice compared with the ApoE^−/−^-AAV mice (Fig. 5 C. D).

## 4 Discussion

Heme oxygenase (HO), a protective and stress-induced protein released from vascular cells, mediates the vascular inflammation in atherosclerosis (Morita, 2005). HO may play an important role in the treatment of atherosclerosis. HO-1 plays a beneficial role in atherosclerosis by increasing antioxidant protection and decreasing the inflammatory response (Orozco et al., 2007). But there are few studies on HO-2 and atherosclerosis. In the study, we found the HO-2 was significantly expressed in ApoE^−/−^ by WD.

Some studies have showed that HO-2 plays a role in inhibition of inflammatory responses (Seta et al., 2006). Our previous research has showed that Heme Oxygenase-2 suppresses TNF-α and IL6 Expression via TLR4/MyD88-Dependent Signaling Pathway in Mouse Cerebral Vascular Endothelial Cells (Chen et al., 2014). The research of Stephen et al. further showed that HO-2 can bind to TRAM, and inhibit the TRAM-dependent LPS-TLR4-induced immune response (Zhu et al., 2017). The expressions of HO-2 in diaphragm and primary macrophages can be induced by LPS (Barreiro et al., 2002; Litvak et al., 2009). In the study, the expression of HO-2 in the IL4-induced peritoneal macrophages was significantly increased compared with the induced-LPS one. Overexpression of HO-2 inhibited the expression of IL-6 and TNFα induced by LPS in cerebral vascular endothelial cells (Chen et al., 2014), whereas knockdown of HO-2 enhanced the expression. These results have indicated that HO-2 acts as a negative feedback regulator in the inflammatory response. Atherosclerosis is a chronic inﬂammatory disease, so we suggest that HO-2 may be involved to inhibit the development of atherosclerosis by macrophages.

The AAV–HO–2 was injected into ApoE^−/−^ on Western diet at 12 weeks. The effect was evaluated at 16 weeks, and the results showed the various indicators of atherosclerosis in the positive mice were significantly higher than in the control mice. Flow cytometry analysis and immunofluorescent staining showed the M2 macrophage (CD206) content in the AAV–HO–2 ApoE^−/−^ were increased compared with the control ApoE^−/−^. The results indicated the HO-2 can participate in the inﬂammatory response by regulating the macrophage polarization. HO-1 is involved in the M2 polarization of macrophages (Weis et al., 2009). Heme oxygenase-2 deletion impairs macrophage function: implication in wound healing (Bellner et al., 2015).

In the study, AAV–HO–2 promoted the reduction of inflammatory factors (Fig. 5), same as our previous research (Chen et al., 2014). The results indicated that HO-2 acts as a negative feedback regulator in inflammatory response, consistent with the higher levels of inflammatory cytokines in an HO-2 KO mouse (Bellner et al., 2009). Further studies may be needed to elaborate the role of HO-2 in regulating the inflammatory response in macrophage, and to uncover HO-2 how to regulate the lipid-modifying proteins.

## 5 Conclusion

In the studies, we observed that increased HO-2 expression correlated with decreased atherosclerotic lesions, proinflammatory cytokines and lipid level in ApoE^−/−^ mice. Further studies are required to elucidate the critical pathways and mechanism involved in the HO-2-mediated protective effects. Our findings demonstrate that HO-2 may be a potential novel therapeutic target for inhibiting or preventing atherosclerotic cardiovascular disease.

## Funding

Jiangsu Key Laboratory of Animal Genetic Breeding and Molecular Design (AGBMD2020001). Innovation and Entrepreneurship Training Program for College Students in Jiangsu Province (202110313073).

## Ethics approval and consent to participate

Animal experimental procedures were performed in accordance with the ethical guidelines of Xuzhou Medical University (Xuzhou, China).

## Tata statement

The datasets used and analyzed during the current study are available from the corresponding author on reasonable request.

## CRediT authorship contribution statement

**Zhenzhen Wang:** Writing – review & editing, Methodology. **Xiaoqiang Zhan:** Methodology, Investigation. **Shuai Yang:** Formal analysis, Resources. **Yang Chen:** Investigation, Resources, Software. **Yingchao Bi:** Conceptualization, Methodology, Investigation, Resources. **Xuemei Xian:** Conceptualization, Methodology, Investigation, Resources, Software. **Quangang Chen:** Investigation, Resources. **Xufeng Han:** Investigation, Resources, Software. **Zhangping Yang:** Supervision, Data curation, Writing – original draft. **Renjin Chen:** Supervision, Conceptualization, Formal analysis, Funding acquisition, Resources, Data curation, Writing – original draft, Writing – review & editing.

## Declaration of competing interest

The authors declare that they have no known competing financial interests or personal relationships that could have appeared to influence the work reported in this paper.

## Data Availability

Data will be made available on request.
