# Supplementary material for: Heme-heme oxygenase-2 reduces the atherosclerosis by preventing inflammation
Source: Curr Res Pharmacol Drug Discov. 2022 Dec 12;4:100141. doi: 10.1016/j.crphar.2022.100141 (PMC9804009; doi:10.1016/j.crphar.2022.100141)
Supplement: Multimedia component 1 [file mmc1.docx]

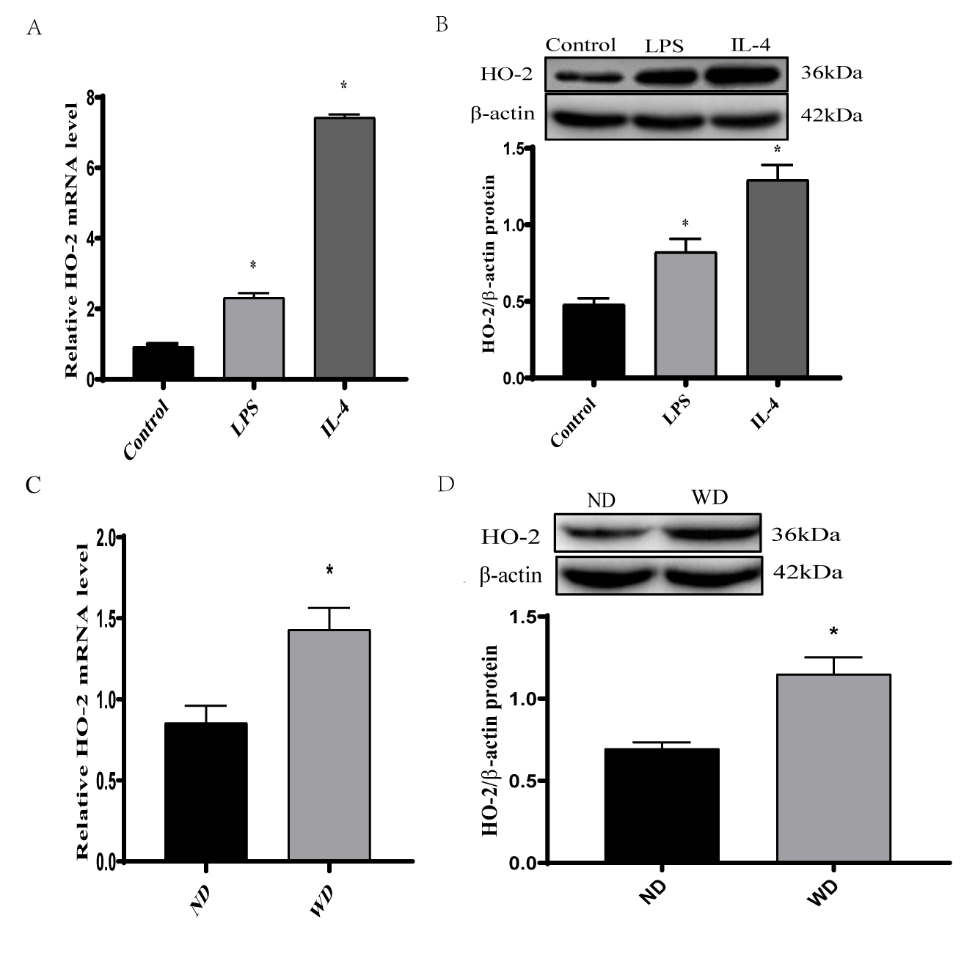


**Fig.** S1 Expression of HO-2 in M1 and M2 macrophage were induced by LPS and IL4, En face aortas of ApoE^-/-^ on Normal diet (ND) and Western diet (WD). A. C, The mRNA expression levels of HO-2 were measured by qPCR (n=3); B. D, The protein expression levels were measured by Western blot (n=3). Data (A-D) are shown as the mean ± SD of two independent experiments performed in triplicate. Values of qPCR and protein expression were normalized to β-actin. * LPS vs control, IL4 vs LPS, *P* <0.05; * WD group vs ND group, *P* <0.05.
